# Improving urban flood resilience via GDELT GKG analyses in China's Sponge Cities

**DOI:** 10.1038/s41598-022-24370-8

**Published:** 2022-11-24

**Authors:** Xiaohui Lu, Faith Ka Shun Chan, Nan Li, Chuke Chen, Wei-Qiang Chen, Hing Kai Chan

**Affiliations:** 1 Key Laboratory of Urban Environment and Health, Institute of Urban Environment, Chinese Academy of Sciences, Xiamen, 361021 China; 2 Nottingham University Business School, University of Nottingham Ningbo China, Ningbo, 315100 China; 3 Xiamen Key Lab of Urban Metabolism, Xiamen, 361021 China; 4 School of Geographical Sciences, University of Nottingham Ningbo China, Ningbo, 315100 China; 5 Water@Leeds, School of Geography, University of Leeds, Leeds, LS2 9JT UK; 6 Research Base for Shenzhen Municipal Policy & Development, Southern University of Science and Technology, Shenzhen, 518000 China; 7 University of Chinese Academy of Sciences, Beijing, 100049 China; 8 Nottingham Ningbo China Beacons of Excellence Research and Innovation Institute, Ningbo, 315100 China

**Keywords:** Natural hazards, Climate-change mitigation

## Abstract

Urban floods are the most severe disaster in most Chinese cities due to rapid urbanisation and climate challenges. Recently, media data analytics has become prominent in enhancing urban flood resilience. In this study, news media data from the GKG of the GDELT project was innovatively used to examine the pattern of news media responses towards urban flooding in China's Sponge City Programme (SCP) pilot cities. We find that public sentiments toward urban flood events have been more positive in SCP pilot cities from 2015 to 2021. News media responses towards urban floods exhibit strong seasonality, which is significantly connected with rainfall patterns. Most of the media articles were posted during the urban flood event. Finally, we suggest the opportunities and challenges in applying GKG data analytics and new technologies for urban flood resilience. The results can provide beneficial references to urban flood management strategies in China's Sponge Cities for associated policymakers and stakeholders.

## Introduction

The latest published IPCC AR6 report indicated that Asian cities, particularly Chinese ones, have experienced severe flood consequences. These cities are affected by frequent cyclone-enhanced surges and intensive rainstorms^1^. More than 157 Chinese cities have suffered from severe urban floods since 2006^2^. Uphill challenges would further exacerbate urban flood risk with large populations and socio-economic assets due to rapid urbanisation and climate change. For example, a 24-h accumulated rainfall of 460 mm hit Beijing on July 21, 2012, leading to a severe urban surface water flood and causing 79 deaths^3^. The urban flood occurred in Zhengzhou on July 20, 2021, with a 1-h rainfall of 202 mm, resulting in the death of 339 people^4^. Urban floods have caused significant impacts on injuries, casualties, and economic losses during the last decade^5,6^. Therefore, enhancing urban flood resilience has become a critical and significant issue for stakeholders to improve the adaptability of the urban systems with increasing urban flood risk^7,8^.

In 2015, the Sponge City Program (SCP) initiated had been conducted for urban flood management. Nature-Based Solutions (NBS) have been used to absorb stormwater as a “Sponge” via restored urban vegetation and soil surface^5^. The SCP is increasing the flood protection standard reaching a 1-in-30-year return period flood event^9^. The SCP has only been initiated for about seven years, which implies the local authorities still need more time to implement and expand the “Sponge” coverage in the urban districts. In recent years, some urban floods have occurred in SCP pilot cities (e.g., Shenzhen 2019, Wuhan 2020, etc.). These urban floods still caused severe consequences and impacts on the local communities and impacted the transport system (roads and subways), public services, and businesses^10^. As urban floods become more frequent and severe, only relying on physical infrastructure could be insufficient to foster flood resilience under uncertainties.

There is a growing popularity for using human-centric information to foster urban flood resilience, with the inspiration from various globally recognised disaster reduction frameworks (i.e., the Sendai Framework Disaster Reduction)^11^. Public responses to urban floods can differ in cities or periods. Public responses are crucial for improving urban flood resilience during preparedness and response^12^. Based on public response analytics, stakeholders and policymakers can make better decisions and co-productions in response to future flood events. Digital media has recently applied to understanding the explicit facts, constraints, and challenges during the past urban flood events^13^. However, most of the existing studies applied social media data (e.g., Weibo, Twitter, Facebook, etc.) at a small scale (e.g., district level)^14^. Existing studies were conducted for some specific urban flood events. It is hard to compare public responses among different cities. The major reason for these gaps can be summarised as digital media data analysis requiring much time and data resources.

Since 2015, building on advances in digital media mining, an inspiring technology that could be transformative in this space, namely, the “*Global Knowledge Graph* (GKG) of the *Global Database of Events, Language and Tone* (GDELT)” project^15^. The GKG contains graph data which is coded from the contents of news media articles from nearly all corners of every country using automated algorithms^16^. It offers various themes to describe the characteristics (e.g., time, emotions, locations) of natural disasters^17,18^. Notably, GKG can overcome a significant challenge in assessing media trends (i.e., access to data). It provides structured data which can be analysed directly. Moreover, news media tends to be more critical and objective than social media because news articles are selected by reporters or editors. Hence, the GKG is a valuable source via media data analytics and useful in urban flood resilience and planning research.

**Figure 1:**
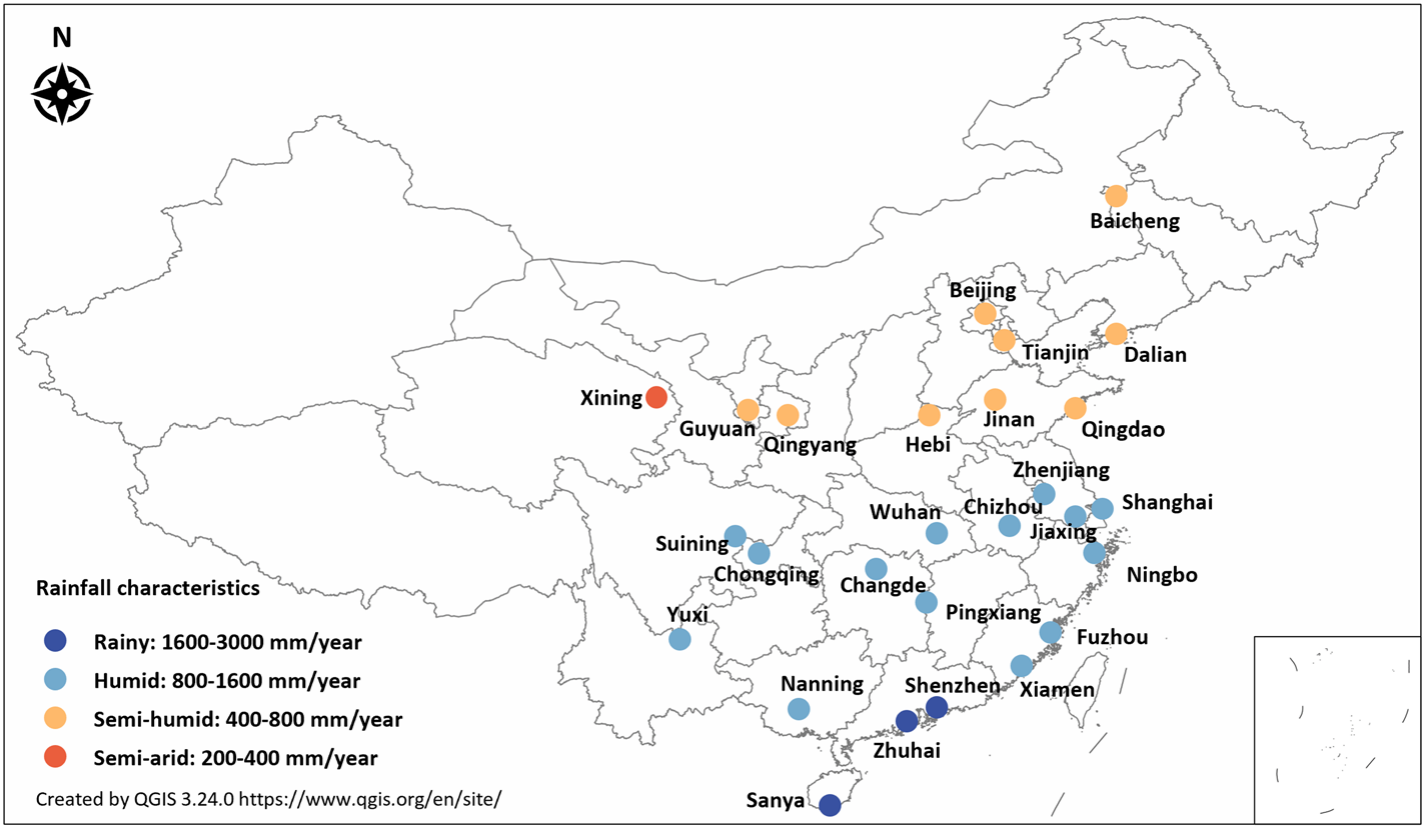
Distribution of SCP pilot cities and rainfall characteristics.

This study aims to answer the research question: how to enhance urban flood risk management and resilience via media data analytics. 27 prefecture-level SCP pilot cities were taken as cases (see Fig. 1). A systematic investigation of news media responses to urban floods is conducted using the GKG data. Firstly, the pattern of media attention and public sentiment is investigated, followed by an investigation of the relationship between news media responses and rainfall. After that, the variation of news media responses during an urban flood event is described. Finally, we discussed the opportunities and challenges in GKG analytics and other new technologies for enhancing urban flood resilience. Through this study, we will contribute to and implement more resilient urban flood management in China's sponge cities and extensively in other Chinese cities.

## Results

### The pattern of news media responses toward urban floods

**Figure 2:**
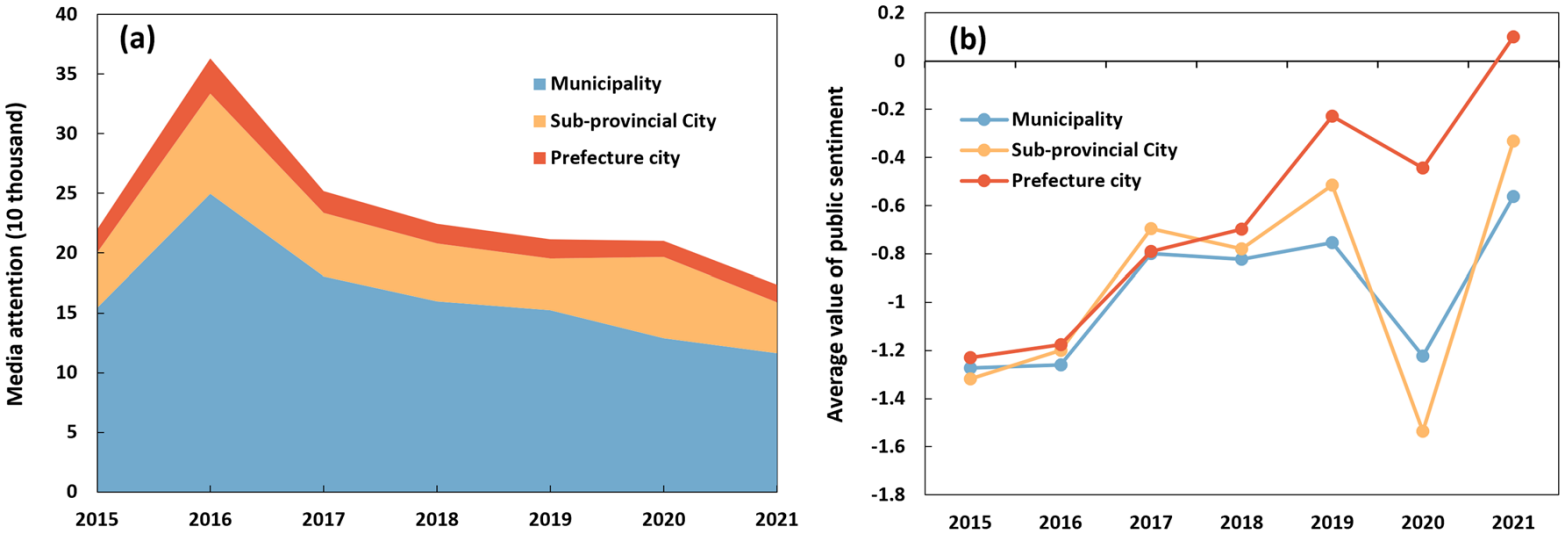
Annual variations of news media responses towards urban floods from 2015 to 2021.

There is a decreasing pattern according to the amount of media articles during the past seven years (see Fig. 2a). On average, there is an annual number of 230 thousand media articles in 27 cities in a year. A total of 360 thousand media articles were released in 2016, which was the highest annual number in the past seven years. There is an increasing trend of positive media articles. The rate of positive media articles has increased substantially from 33.8% in 2015 to 46.1% in 2021.

It may be attributed to the implementation of the first SCP objective (i.e., greater than 20% area with the volume capture ratio > 70%)^9^. During the first three years of the establishment of the SCP practice, the Chinese National and Local Municipality Governments have made great efforts to reduce surface water runoff. Grey (e.g., floodwalls, drainage facilities, water pumps) and green infrastructures (e.g., green roofs, permeable pavements, urban forests) are integrated to enhance flood resilience in China's Sponge Cities.

The annual value of public sentiment has increased from − 1.28 in 2015 to − 0.45 in 2021. The lowest average annual public sentiment appeared in 2020 (i.e., − 1.27). It may be attributed to the severe urban floods in Wuhan, Chongqing, Beijing, and Chizhou. For example, we extracted the records of five heavy rainstorms that hit cities along the Yangtze River Basin in July 2020. The average rainfall is 259.6 mm, the highest in July since 1961. As a result, the consequences caused 99 deaths, 36,000 house collapses, and 132 billion RMB direct economic losses across 11 provinces, e.g., Anhui, Hubei, and Chongqing Provinces.

The differences in news media responses were noticeable among municipalities, sub-provincial cities, and prefecture cities (see Fig. 2). The most media articles were released in the four municipalities (i.e., Beijing, Shanghai, Tianjin, and Chongqing), accounting for approximately 69% of total media articles (see Fig. 2a). The number of media articles in 7 sub-provincial cities (including Jinan, Wuhan, Xiamen, Dalian, Ningbo, Qingdao, and Shenzhen) is more than 384 thousand. Sixteen prefecture-level cities get relatively rarer media articles, accounting for 7% of total media articles. Public sentiment in prefecture-level cities is higher than in municipalities and sub-provincial cities (see Fig. 2b). The average annual value of public sentiment in municipalities, sub-provincial, and prefecture-level cities are − 0.956, − 0.911, and − 0.638, respectively.

The reason for higher media attention to urban floods may be the outdated drainage facilities that cannot support the fast-urban transformation in municipalities and sub-provincial cities. Rapid urbanisation has led to a significant increase in the impervious surface area (e.g., roads, malls, etc.) in municipalities and sub-provincial cities since the “open-door policy” in 1978^19^. For example, Shenzhen's road density was 9.5 km/km^2^ in 2020^20^. The current drainage facilities can cope with runoff caused by 1-in-10 year or even 1-in-1-year return period flood events in China's municipalities and sub-provincial cities, especially in the old town areas. Additionally, rising media attention can be attributed to the fast development of digital media in cities with higher economic development and urbanisation levels. Moreover, Beijing, Shanghai, and Shenzhen are ranked as the top-tier cities (i.e., Alpha cities—the most important cities in China) worldwide. The information exchangeability and sci-technology innovation ability are noticeably high in these cities^21^.

Media attention is concentrated in densely populated cities, including Shanghai (258,811), Shenzhen (127,938), Tianjin (118,141), Wuhan (94,675), and Chongqing (90,759). As for the prefecture-level cities, media attention in Eastern China's cities (e.g., Fuzhou, Ningbo, Xiamen) is higher than that in Central (e.g., Baicheng, Chizhou, Pingxiang, Hebi, and Changde) and Western China's cities (e.g., Guyuan, Xining, Qingyang, and Suining). It is interesting to highlight that Zhuhai and Sanya locate in the Southern coastal cities, but their number of media articles is only 3935 and 6461, respectively. It is related to the low frequency of severe urban floods caused by rainstorms. Beijing (the capital city of China) has gotten the highest media attention on urban floods, but the value of the public sentiment is low (− 1.194). Notably, the phenomenon is different in Shanghai and Shenzhen. Shanghai and Shenzhen have relatively high media attention and public sentiment, with public sentiment values of − 0.729 and − 0.664, respectively. The highest value of public sentiment exists in Jiaxing, Suining, Qingyang, and Sanya, with − 0.197, − 0.259, − 0.311, and − 0.316, respectively. Wuhan has the lowest average value of public sentiment (i.e., − 1.807) due to frequent extreme rainstorms and induced urban floods. For example, a heavy rainstorm hit Wuhan on June 23, 2015, still leading to a 67 million RMB economic loss.

**Figure 3:**
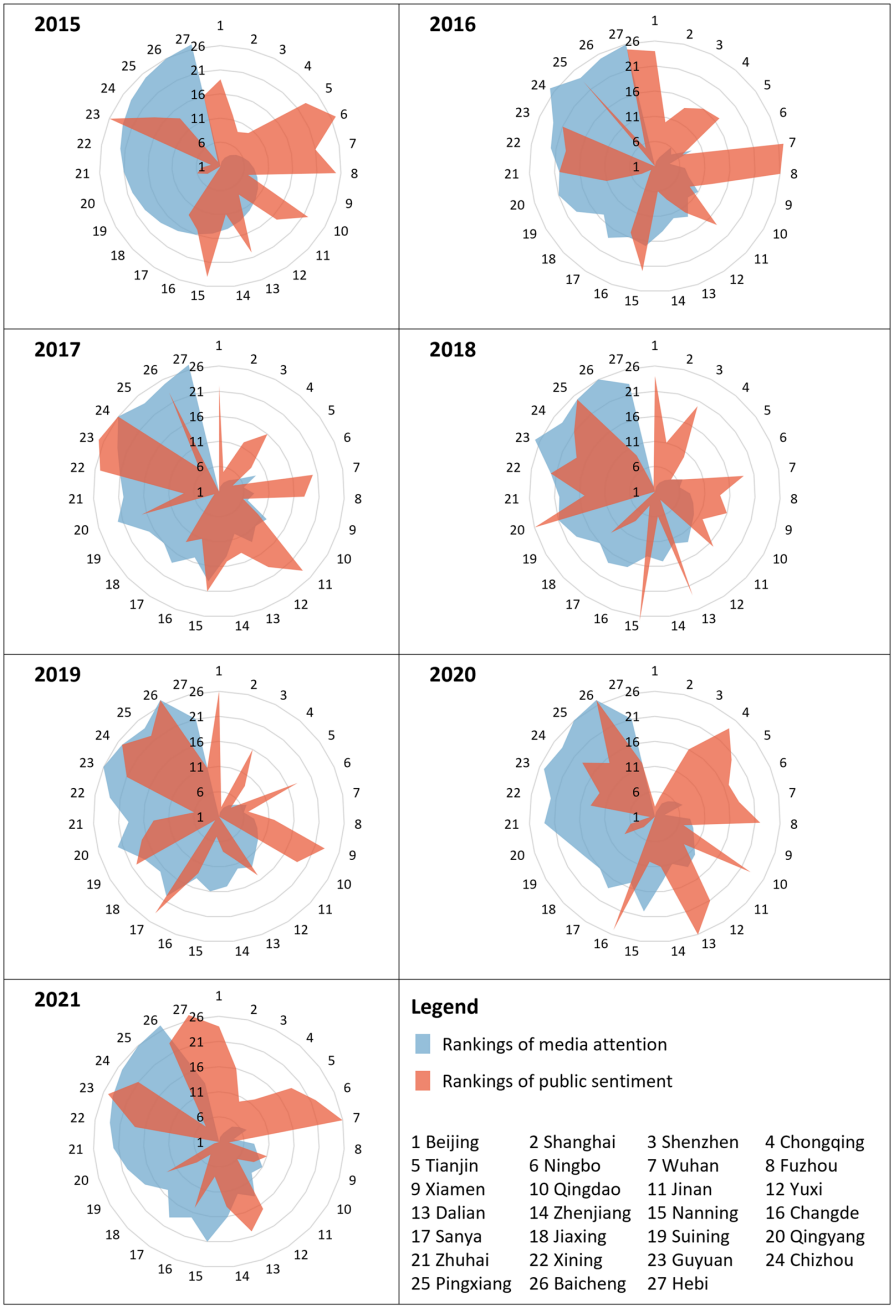
Annual variations of news media responses towards urban floods in different cities from 2015 to 2021.

Our findings show no significant changes in the ranking of media attention from 2015 to 2021 (Fig. 3). Beijing, Shanghai, Shenzhen, Chongqing, and Tianjin have made essential contributions according to the number of media articles. The most significant change was at Hebi, which climbed 14 spots from 27th in 2015 to 13th in 2021. This increase corresponds to the rainstorm in Henan province during July 17–22, 2021, leading to a 1.18 million flood-hit population (i.e., accounting for 65.1% of the total population in Hebi) and a 1.77 billion RMB direct economic loss.

Public sentiment ranking has been constantly in flux in the past seven years (Fig. 3). There were dramatic changes (about 25 or 26 spots changed) in public sentiment ranking exhibits in cities with fewer media articles, including Qingyang, Zhuhai, Guyuan, Chizhou, and Hebi. This phenomenon may be due to a change in rainfall that is still tiny relative to rainfall variability in China^22^. In other words, extreme rainfalls increase while light/mild rainfalls decrease. Public sentiments are polarised in Hebi, with the 1st rank and 27th rank in 2017 and 2021, respectively. The public sentiment has been steady and could get higher in Xiamen, Sanya, and Jiaxing from 2015 to 2021.

**Figure 4:**
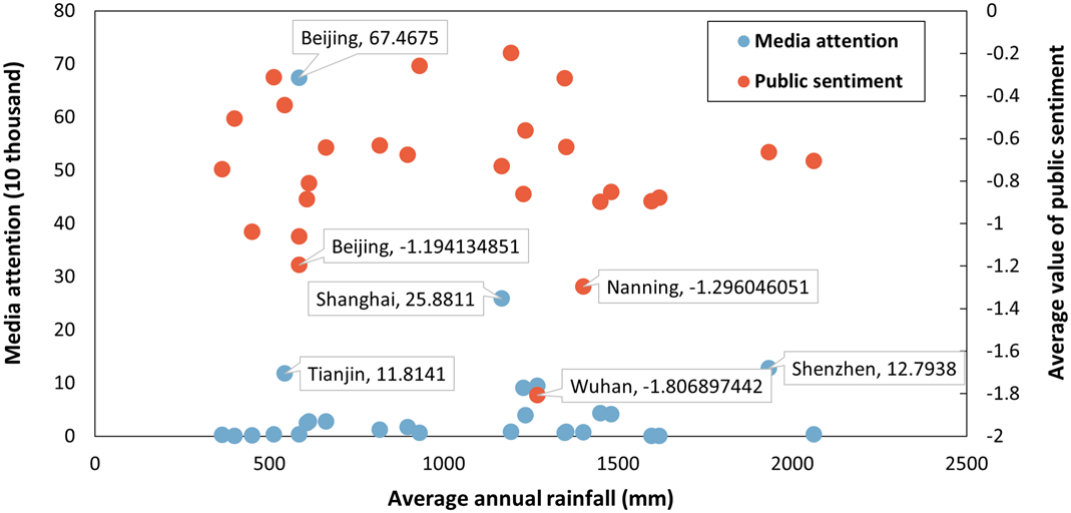
The relationship between news media responses towards urban floods and average annual rainfall in SCP pilot cities.

The top cities ranked by media attention may not have high public sentiment. According to the media attention, Beijing has been the top 1 city, whereas the public sentiment is significantly low (see Fig. 4b) from 2015 to 2021. The top values of public sentiment are − 0.193 (Jiaxing, 2015), − 0.301 (Chizhou, 2016), − 0.092 (Hebi, 2017), − 0.046 (Suning, 2018), 1.505 (Hebi, 2019), 0.705 (Qingyang, 2020) and 1.715 (Zhuhai, 2021).

### The relationship between news media response and rainfall

Neither media attention nor public sentiment is significantly associated with cities having a higher average annual rainfall (see Fig. 4). For example, the number of media articles in Chizhou (1158) and Pingxiang (1165) is significantly low, whereas the average annual rainfall in Chizhou and Pingxiang is 1618 mm and 1596 mm, respectively. The average annual rainfall in Beijing (585 mm), Hebi (586 mm), and Guyuan (451 mm) are lower than that in most other areas. However, the public sentiment in these three cities was significantly negative, with − 1.194 in Beijing, − 1.061 in Hebi, and − 1.038 in Guyuan. Besides, Wuhan has the lowest public sentiment, whereas the average annual rainfall in Wuhan is about 1269 mm, ranked 10th in all pilot cities. Zhuhai, Shenzhen, and Sanya stand out among all pilot cities with their high average annual rainfall and high value of public sentiment.

**Figure 5:**
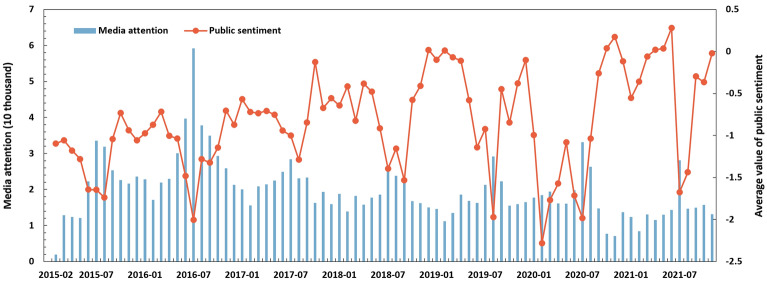
Monthly variations of news media response towards urban floods from 2015 to 2021.

News media responses to urban floods exhibit strong seasonality (Fig. 5). The number of media articles in summer is higher (about 1.4–1.8 times) than in other seasons. Indeed, the monthly distribution of media articles is similar to the monthly rainfall distribution, concentrating from May to September (i.e., the wet season). The peak of media articles is in July almost every year from 2015 to 2021. In particular, the number of media articles exceeded 50 thousand in July 2016. In the term of monthly public sentiment, the lowest values were recorded in August 2015 (− 1.74), July 2016 (− 2.00), August 2017 (− 1.29), September 2018 (− 1.53), August 2019 (− 1.97), February 2020 (− 2.28) and July 2021 (− 1.67). On contrary, the positive sentiments were recorded in December 2018 (0.02), February 2019 (0.01), October 2020 (0.04), November 2020 (0.17), April 2021 (0.02), May 2021 (0.04), June 2021 (0.28), and December 2021 (0.46).

**Figure 6:**
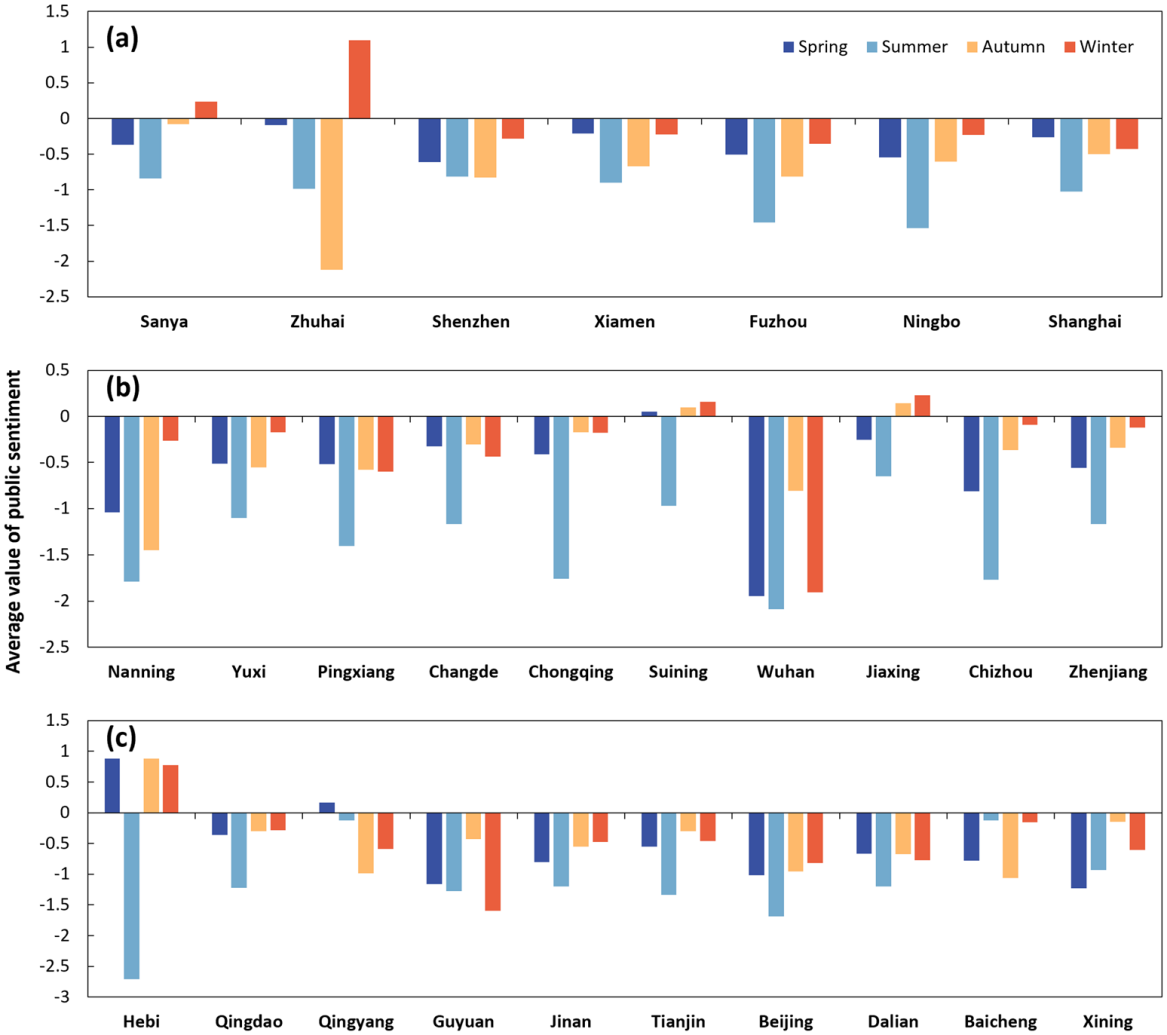
Average seasonal value of public sentiment to urban flood in coastal rainy or humid cities (**a**), inland humid cities (**b**), and semi-arid cities (**c**).

Consistent findings have been found in the overall pattern of 27 pilot cities. Public sentiment in each city is also seasonal (Fig. 6). In spring, public sentiments in Hebi, Qingyang, and Suining are positive, with an average tone of 0.879, 0.165, and 0.050, respectively. The lowest average tone exists in Wuhan (− 1.946), followed by Xining (− 1.233) and Guyuan (− 1.163). In the summer season, the public sentiments in all cities are negative, with a minimum value of − 2.712 (Hebi) and a maximum value of − 0.128 (Qingyang). In Autumn, Hebi, Jiaxing, and Suining have the highest average tone, with 0.879, 0.143, and 0.097, respectively. Zhuhai has the lowest average tone (− 2.123). In winter, there are five cities (including Zhuhai, Hebi, Sanya, Jiaxing, and Suining) with positive public sentiments. The most negative city is Wuhan, with an average tone of − 1.904.

### The variation of news media response during an urban flood event

**Figure 7:**
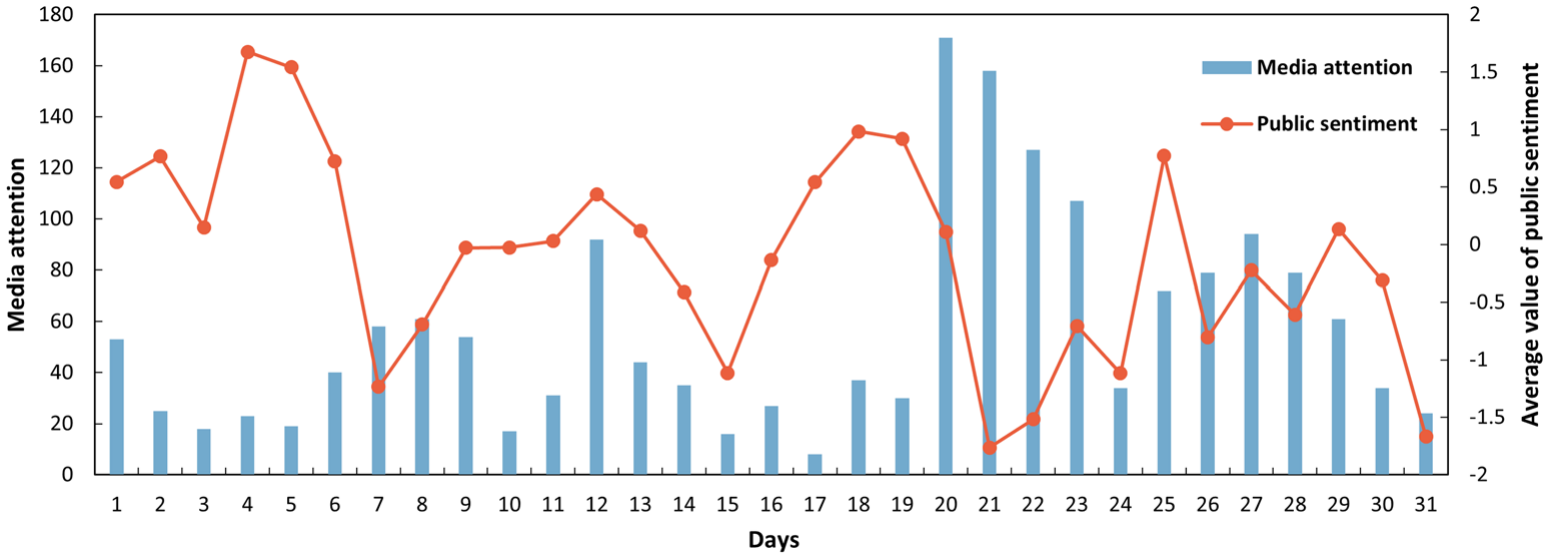
Example of daily news media responses towards urban floods, i.e., heavy rainstorms in Hebi in July 2021.

Most of the media articles were posted during the urban flood event. Figure 7 juxtaposes the daily variations of news media response to urban floods in July 2021. The extreme rainstorm in Hebi on July 17–22, 2021, was an example of the daily variation of news media response to urban floods. According to the official records, the maximum hourly rainfall is 51.1 mm, and the rainfall accumulation is about 569.4 mm (nearly annual rainfall) during the five days^23^. The peak of media attention appeared on July 20 (171), whereas the total number of media articles was only 75 during July 17–19 in 2021. The most negative public sentiment exhibited on July 21 (− 1.76) was due to the torrential rainstorm that occurred during 20:00–23:00 on July 21, with maximum hourly rainfall of 51.1 mm. The number of media articles was 107 on July 23, the first day after a heavy rainstorm.

Such findings further justified that media attention and public sentiment tend to peak and be significantly negative during urban flood events due to people's daily life being directly affected^11,13^. The results reflected that the public had not prepared for upcoming flooding. Indeed, more warning information should be posted to address mental and physical preparations before urban floods. Local Municipality authorities might provide more information on warning and related meteorological information, integrating early warning signals with the consequence of previous urban floods (e.g., traffic disruption, car flooding). The consequence of previous urban floods may enhance public awareness of urban flood preparation.

## Discussion

Climate change and urbanisation will exacerbate urban flood risk in the next few decades. Over the past 50 years, there has been a 10% increase in the number of rainstorm days in China^24^. According to the latest published IPCC AR6 Report, a 1-in-100 years sea-level event could become more frequent and occur commonly in Southern China^25,26^. From the surface water flooding (waterlogging) issue, more developed cities face more severe surface water floods in which the peak runoff is suffocated on top of the land surfaces (i.e., roads, cycle lanes, and pedestrians). In these Chinese cities, urbanisation (i.e., urban development) and construction-led built areas have dramatically replaced numerous aquatic ecosystems (e.g., marshland, lakes) and artificial water systems. Most of the current drainage systems can only cope with surface runoff caused by 1-in-1-year return period flood events, which are insufficient to relieve the urban peak discharge and offload the surface runoff from road networks in many Chinese cities^1^.

Urban flood management practices are required not only via engineering approaches (e.g. the construction of grey and green infrastructures) but also to explore human-centric information to improve preparedness and responses. Digital media platforms (e.g., news media, social media) and the database we illustrated in this study (i.e., GDELT GKG) are significantly helpful for citizens to update real-time information on waterlogging depth and service disruption^27^. Communities and stakeholders can be well informed. This study confirms that digital media can provide valuable information on public responses to urban floods for further analyses and understanding by local governments regarding flood prevention, preparedness, response, and recovery. The results tend to be more critical and objective via news media data than social media. The GKG shows strong potential for investigating public responses to urban floods and other disasters or events as it monitors and analyses web-based articles from around the world. Based on the knowledge gained from this study, the application of GKG analytics and other new technologies are discussed for enhancing urban flood resilience.

Firstly, stakeholders and policymakers can adopt GKG data analytics to design more effective urban flood preparedness and responses. The analysis of variations of news media responses to urban floods during an urban flood event further confirms that the public has not been well prepared and forewarned for potential disruptions caused by urban floods^11^. Moreover, citizens are not significantly sensitive to early warning signals (e.g., red rainstorm warnings) issued by the Meteorological Bureau. More warning information should be posted for better preparation before the urban flood events. Meanwhile, the GKG approach can help stakeholders and policymakers track what happened before, during, and after the urban flood.

Moreover, GKG data analytics can provide a novel perspective for estimating the effectiveness of policy implementations. According to our findings, from the perspective of news media responses to urban flooding, the SCP has improved urban flood resilience in 27 pilot cities in China. After the first stage (the first 3-year from 2015 to 2018) of SCP pilot experiments, most pilot cities could cope with an accumulation of rainfall-enhanced stormwater discharge during the rainstorm event (at low to moderate rainfall up to 1-in-30 years storm events). However, the urban surface flood issues are still significantly severe during an extreme rainstorm (e.g., 1-in-100 years urban flood event). Public responses to urban flood events are becoming positive in SCP pilot cities from 2015 to 2021. The results show that less than half of the SCP pilot cities presented negative sentiments towards flooding events in 2021, whereas public sentiments were negative in all cities in 2015. Based on the estimation of policy implementation effectiveness, stakeholders and policymakers can craft policies that make vulnerable cities and communities more resilient in the future.

Finally, it is critical to incorporate news media analytics with other new technologies to enhance urban flood resilience. Under the progress and vision of SCP, further integration and application of various technologies would provide a more substantial basis for stakeholders to make better decisions^28^. Before urban flood events, the Geographic Information System and Remote Sensing can be used to simulate runoff generation and depth under various rainfall scenarios alongside some established stormwater modelling tools (i.e., via the SWMM, InfoWorks, etc.)^29^. It would indicate the appropriate vulnerable locations and the possible harm flooding could create. Based on the simulation results, city authorities can provide rainstorm warning signals and related urban flooding risk signals before the rainstorm. Moreover, municipality authorities can illustrate the urban flood consequences caused by previous rainstorms while improving the warning system for impending rainstorms.

Information technologies (e.g., news media, social media, 3D mapping services, Internet of Things) are encouraged to enhance better urban flood response during urban flood events. For example, using media data and technologies for better preparation and prevention noticeably reduced flood impacts in Ningbo during severe typhoon *In-Fa* event 2021^30^. Indeed, *In-Fa* caused a 150–200 mm/24 h rainstorm and severe surface water flooding in Ningbo, but successful preparation, response, and recovery ensured “zero causalities” and reduced substantial economic losses^31^.

Furthermore, the Internet of Things and video surveillance systems may be considered to monitor drainage systems and surface runoff during a rainstorm event^11^. The emergency department should take timely response measures according to real-time monitoring information. Meanwhile, the monitoring results can validate and improve the simulation results. Social media also assists in emergency response (e.g., police and fire forces, ambulances, civil aid services, and medical services)^32^. With more applications of new technologies, local governments may develop volunteer platforms for citizens to update the flooding information. Public participation is vital in SCP implementation and flood resilience enhancement, e.g., project planning and green finance fund raising^33^.

Although creating tools or databases like GKG is a significant step forward for enhancing urban flood resilience, these tools or databases come with biases that must be understood. The accuracy of GKG location tagging is not foolproof. For example, the field “*location*” shows the flood event that occurred in Zhuhai, but the field “*longitude*” and field “*latitude*” shows the flood event that occurred in Shenzhen. Based on the data accuracy verification, we found that the fields “longitude” and “latitude” are correct. Hence, we have used QGIS for data pre-processing to ensure the accuracy of record location before data analytics. Moreover, GKG data has an unequal distribution geographically due to the different media environments and technology. Media environment and technology should be considered in media attention analytics.

## Conclusion

In conclusion, this study investigates the patterns of public responses to urban flooding events in China's Sponge Cities based on analysing news media data from the GDELT GKG. It represents a modest attempt to improve our understanding of urban flood resilience. The findings reveal that public responses toward urban flood events are becoming positive in SCP pilot cities from 2015 to 2021. After the medium-term SCP construction (post the 2020s), most of the pilot Sponge cities could cope with an accumulation of rainfall during a rainstorm of low to moderate rainfall to show the SCP is gradually addressing the urban surface flood and stormwater issues. Still, some Chinese cities are suffering urban floods from extreme rainstorms.

In this study, we have found that: (1) High media attention towards urban floods was found in cities with dense populations and rapid development. (2) Public sentiments have been dramatically changing yearly, whereas annual rainfall has no obvious changes. (3) Media responses towards urban floods exhibit strong seasonality, mainly concentrating on the summer and autumn seasons (due to rainfall patterns in China). This study shows that the public attitude towards urban floods is negative in summer, especially in humid inland cities. (4) Most media articles were posted during the urban flood event.

Hence, this study recommended the application of new technologies (e.g., GDELT GKG) for improving flood resilience. That will help enhance knowledge transformation among stakeholders, particularly from the previous flood events and experiences (e.g., reflections from communities and media). In future studies, incorporating social-economic data will be helpful for a better understanding of preparedness and response disparities of urban flood resilience. Besides, as the GKG is a graph model over the entire world, more true insights into urban floods can be captured via graph analytics, such as the government networks during the urban flood response. That can improve the local authorities and decision-makers' well-informed about the ongoing situation.

## Methods

### Study areas

27 SCP pilot prefecture-level cities were selected as cases for this study. The first batch of nominated pilot cities includes Zhenjiang, Jiaxing, Chizhou, Xiamen, Pingxiang, Jinan, Hebi, Wuhan, Changde, Nanning, Chongqing, and Suining. The second batch of nominated pilot cities includes Beijing, Tianjin, Dalian, Shanghai, Ningbo, Fuzhou, Qingdao, Zhuhai, Shenzhen, Sanya, Yuxi, Qingyang, Xining, and Guyuan. The average annual rainfall varies in different cities, ranging from 365 mm in Xining to 2061 mm in Zhuhai. The volume capture ratio of annual rainfall is the primary control index for SCP implementation, which is required to reach 80–85% after the completed construction of SCP^28^. The volume capture ratio of annual rainfall and meteorological conditions thus varies across different cities in China (see Table 1). In the three rainy cities (i.e., Shenzhen, Zhuhai, and Sanya), the volume capture ratio of annual rainfall is 60–85%, whereas the value is 80–90% in Baicheng, Yuxi, Qingyang, Xining, and Guyuan. The volume capture ratio of annual rainfall is 70–85% in most SCP pilot cities.

**Table 1:** Volume capture ratio of annual rainfall in SCP pilot cities^9,34^.

Region	VCR of annual rainfall	Pilot cities
Region I	85–90%	Qingyang, Xining, Guyuan
Region II	80–90%	Baicheng, Yuxi
Region III	75–85%	Chongqing, Jiaxing, Chizhou, Pingxiang, Changde, Suining, Beijing, Tianjin, Shanghai, Ningbo, Fuzhou
Region IV	70–85%	Jinan, Wuhan, Xiamen, Zhenjiang, Hebi, Nanning, Dalian, Qingdao
Region V	60–85%	Shenzhen, Zhuhai, Sanya

### GDELT GKG

Flood knowledge graph data was obtained from the GDELT GKG (https://blog.gdeltproject.org) via Google BigQuery. The GKG shares real-time information and metadata with the world by cataloguing world events and their latent dimensions, geographic characteristics, and network structures. This codified metadata (but not the text of the articles) is then released as an every 15-min open data stream since 2015. It includes broadcast, print, and online news sources, incorporating more than 100 live translated languages. The GKG contains a massive-scale network of inter-connected events, dates, locations (i.e., countries, states/provinces, and districts), themes (i.e., topics), tones (i.e., emotions), and sources (i.e., URLs). Each GKG record is a unique pairing of a set of names (events, counts, dates, actors, locations, themes, and tones) and a set of media articles in which the set of names appears. GKG theme indicates discussions around 15 GKG themes are related to flood, such as "NATURE_DISASTER_FLOOED_ROAD".

### Data analytics

**Figure 8:**
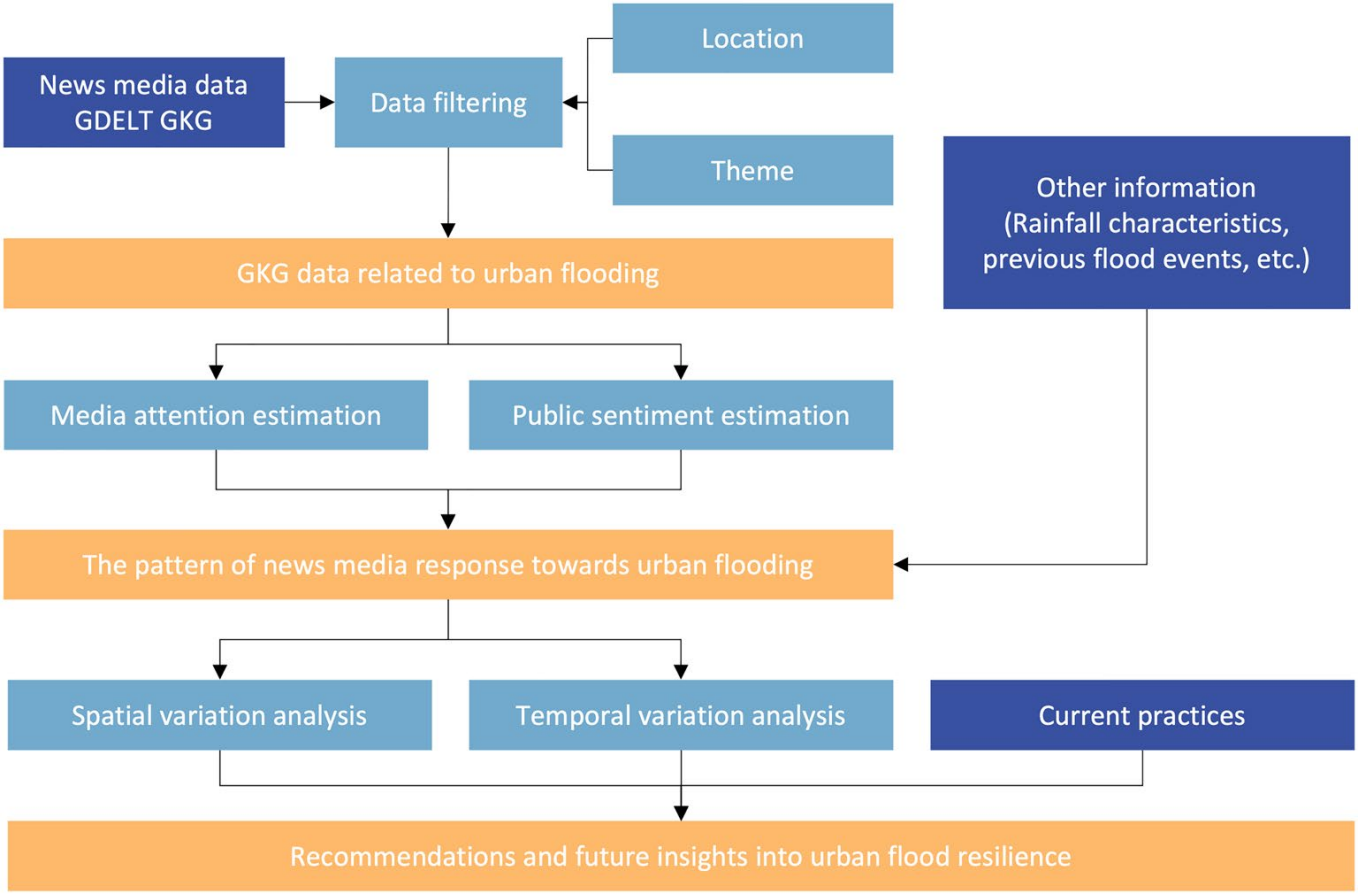
Research Framework.

Media attention and public sentiment were analysed in each city and time interval. Figure 8 shows the processing steps of GKG data analytics for urban flood resilience.

- *Data filtering* This study collected complete GKG data over the past seven years (i.e., from 2015 to 2021). Location and theme were used to identify whether a GKG record was related to urban flooding in 27 SCP pilot cities. 1,655,385 pieces of GKG records were identified as flooding-related in 27 SCP pilot cities.
- *Media attention estimation* Media attention refers to the number of media articles about floods in a city (or during a year/month/day interval). Media attention represents the extent of public concern about urban flooding.
- *Public sentiment estimation* Public sentiment refers to the average tone of articles across all media about floods in a city (or during a year/month/day interval). Media tone is calculated as a positive score (i.e., percentage of all words with a positive emotional connotation) minus a negative score (i.e., percentage of all words with a negative emotional connotation) by the GDELT project. The media tone ranges from − 100 (extremely negative) to + 100 (extremely positive), and common values range between − 10 and + 10. Public sentiment represents public emotional responses towards urban flood in a city (or during a year/month/day interval), including negative (i.e., media tone < 0), neutral (i.e., media tone = 0), and positive (i.e., media tone > 0)^35^.
- *Temporal and spatial variation analysis* Other data (e.g., rainfall characteristics and previous flood events) were incorporated with GKG data for temporal and spatial variation analysis. Temporal variation analysis was adopted to examine media responses towards urban flooding in different periods, including yearly variation analysis, monthly variation analysis, and variation analysis during an urban flood event (i.e., daily variation). Spatial variation analysis was adopted to compare media responses to urban flooding in different cities. Meanwhile, current practices were incorporated with the temporal and spatial variation analysis results to provide recommendations and future insights.

## Data Availability

The data that supports the findings of this study are available from correspondent authors upon reasonable request.
